# Molecular Interactions that Enable Movement of the Lyme Disease Agent from the Tick Gut into the Hemolymph

**DOI:** 10.1371/journal.ppat.1002079

**Published:** 2011-06-09

**Authors:** Lili Zhang, Yue Zhang, Sarojini Adusumilli, Lei Liu, Sukanya Narasimhan, Jianfeng Dai, Yang O. Zhao, Erol Fikrig

**Affiliations:** 1 Section of Infectious Diseases, Department of Internal Medicine, Yale University School of Medicine, New Haven, Connecticut, United States of America; 2 Howard Hughes Medical Institute, Chevy Chase, Maryland, United States of America; National Institute of Health, United States of America

## Abstract

*Borrelia burgdorferi*, the causative agent of Lyme disease, is transmitted to humans by bite of *Ixodes scapularis* ticks. The mechanisms by which the bacterium is transmitted from vector to host are poorly understood. In this study, we show that the F(ab)_2_ fragments of BBE31, a *B.burgdorferi* outer-surface lipoprotein, interfere with the migration of the spirochete from tick gut into the hemolymph during tick feeding. The decreased hemolymph infection results in lower salivary glands infection, and consequently attenuates mouse infection by tick-transmitted *B. burgdorferi*. Using a yeast surface display approach, a tick gut protein named TRE31 was identified to interact with BBE31. Silencing *tre31* also decreased the *B. burgdorferi* burden in the tick hemolymph. Delineating the specific spirochete and arthropod ligands required for *B. burgdorferi* movement in the tick may lead to new strategies to interrupt the life cycle of the Lyme disease agent.

## Author Summary

Lyme disease, the most common tick-borne illness in North America, is caused by *Borrelia burgdorferi*. Currently, spirochete and tick molecules that facilitate *Borrelia* migration within the vector, a key step for mammalian infection by tick-transmitted spirochetes, have not yet been identified. In this study, we show that F(ab)_2_ fragments of BBE31, a *B.burgdorferi* outer-surface lipoprotein, interfere with the spirochete migration from the tick gut into the hemolymph. Our results indicated that decreased hemolymph infection by blocking BBE31 resulted in lower salivary glands infection, which eventually attenuated murine infection by tick-transmitted *B.burgdorferi*. We also found that a tick gut protein TRE31 enables *Borrelia* movement by interacting with BBE31. This finding provides novel insights into the transmission of spirochete within the vector and provides potential vaccine targets to block the microbial life cycle within the vector.

## Introduction

*Borrelia burgdorferi* is the causative agent of Lyme disease, the most common tick-borne illness in the United States and selected regions of Eurasia [1], [2]. The spirochete is maintained in an enzootic life cycle, which involves both tick and vertebrate host [2]. *B. burgdorferi* transmission to humans occurs predominantly by nymphal *Ixodes scapularis* [3]. Previous work has identified some *Borrelia* outer-surface lipoprotein genes induced during nymphal feeding, such as *ospC*, *bbk32*, *bba64* and *bba07* [4], [5], [6], [7], [8]. Because these genes were identified using whole ticks as the template, their detailed expression profile in different tick tissues has not been investigated.

*B.burgdoferi* is restricted primarily to the gut of unfed ticks. After tick feeding commences, spirochetes multiply rapidly in the gut and disperse through the hemolymph into the salivary glands where they are transmitted to the host via expelled saliva [9]. Host molecules can help the spirochete survive in different environments by interacting with different *Borrelia* lipoproteins. For example, a tick gut receptor, TROSPA, interacts with *Borrelia* outer-surface protein A (OspA), facilitating the spirochete to colonize the tick gut [10]. Tick salivary gland protein Salp15 can bind to OspC to protect spirochetes from antibody-mediated killing in the early stage of murine infection [11]. DbpA, DbpB or BBK32 bind to host extracellular matrix proteins like decorin or fibronectin, mediating the tissue adherence of *Borrelia* in the initial stage of Lyme disease [12], [13], [14]. At present, spirochete and tick molecules that facilitate *Borrelia* migration within the vector, a key step for mammalian infection by tick-transmitted spirochetes, have not yet been identified.

## Results

### *bbe31* preferentially expressed in fed nymph

*B.burgdorferi* contains more than 150 lipoproteins, many of which contribute to the bacterial outer-surface and play important roles throughout the spirochete life cycle. To identify lipoproteins required for *Borrelial* tick-to-host transmission, we measured expression of all these putative lipoprotein genes in unfed tick gut, fed gut and salivary glands through q-RT-PCR (Unpublished data). *bbe31*, expression was greatly induced in fed gut and markedly declined in the salivary glands, was therefore selected for further study as a potential gene important in spirochete migration in the tick.

**Figure 1:**
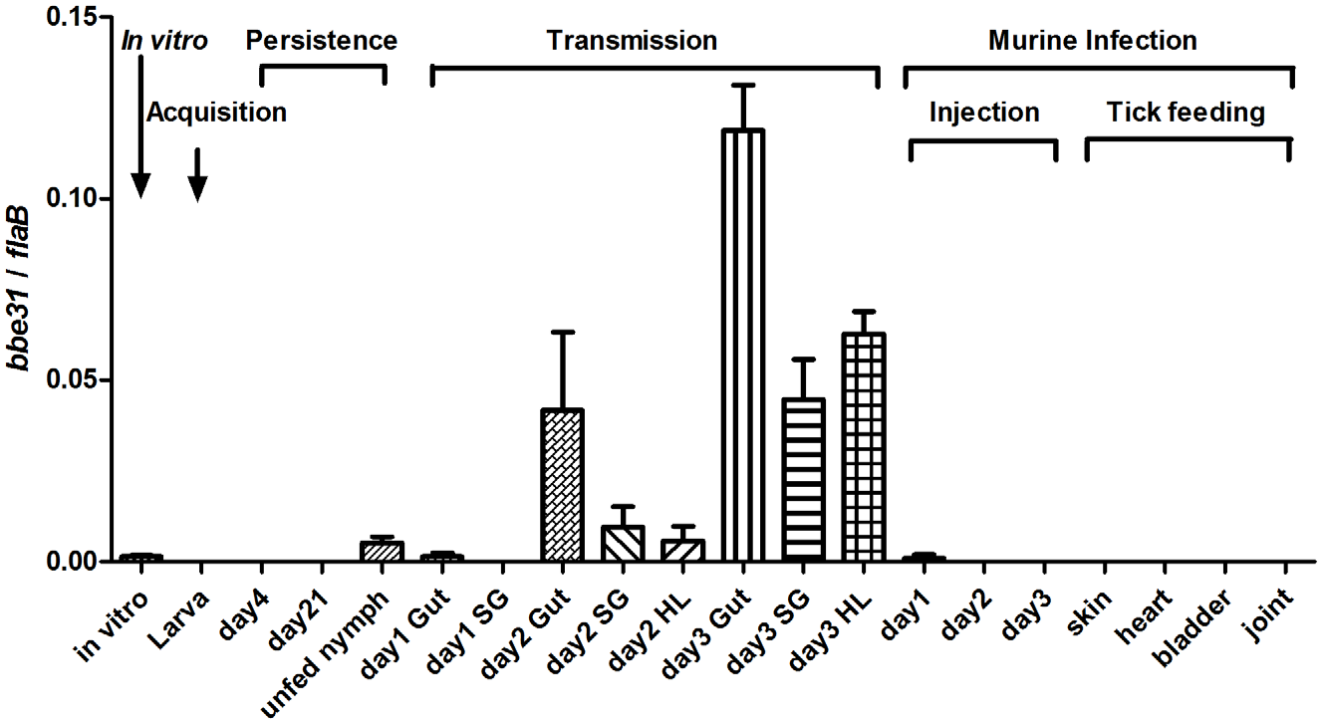
*bbe3*1 expression profile throughout representative stages of the natural spirochete life cycle. *bbe31* is preferentially expressed in fed tick. It is induced from the 2^nd^ day, expressed in all the tested tissues including gut, hemolymph and salivary glands, and showed the highest expression level in the fed gut. Both mean and SD were calculated from 2 independent experiments, with 3 mRNA samples each experiment. *In vitro*, *B.burgdorferi* N40 grown in BSK medium with a concentration of 1×10^7^/ml; Larva, *I.scapularis* larva fed on N40-infected mouse till engorged; day4, larva 4-day after feeding; day 21; larva 21-day after feeding; day1 Gut, day2 Gut and day3 Gut, guts of N40-nymphs feeding on C3H mice for 24, 48 and 72 hours; SG, salivary glands; HL, hemolymph; Injection, mouse inoculated with 1*10^6^ *B.burgdorferi* N40; day1, day2 and day3, mouse localized skin 24, 48 or 72 hours after *B.burgdorferi* N40 infection through needle inoculation; skin, heart, bladder and joint, mouse tissues collected 21 days after N40-infected nymphs feeding on C3H mice.

Detailed expression profiling is useful for investigations of the potential location for gene function. In this study, we measured *bbe31* expression throughout representative stages of the natural spirochete life (Figure 1), including the spirochete acquisition by larva when feeding on *B.burgdoferi* infected-C3H mice (Acquisition), persistence through the molting period (Persistence), transmission from infected nymph to clean C3H mice during nymph feeding (Transmission), survival from the host innate immune system (Injection) and successful infection of several murine host tissues (Tick feeding). We found that *bbe31* is not expressed during larva acquisition and the molting period. It is also not expressed in the murine host. The gene is expressed at very low level *in vitro* and in unfed nymph. Compare to the expression in unfed nymph, *bbe31* is greatly induced during tick feeding. Although expression was induced in all the three tested tissues including tick gut, hemolymph and salivary glands from the 2^nd^ day of feeding, the highest expression level was seen in tick gut. This suggests that BBE31 may play a role during *B.burgdoferi* transmission from tick to the murine host.

### BBE31 is an outer-surface exposed protein

**Figure 2:**
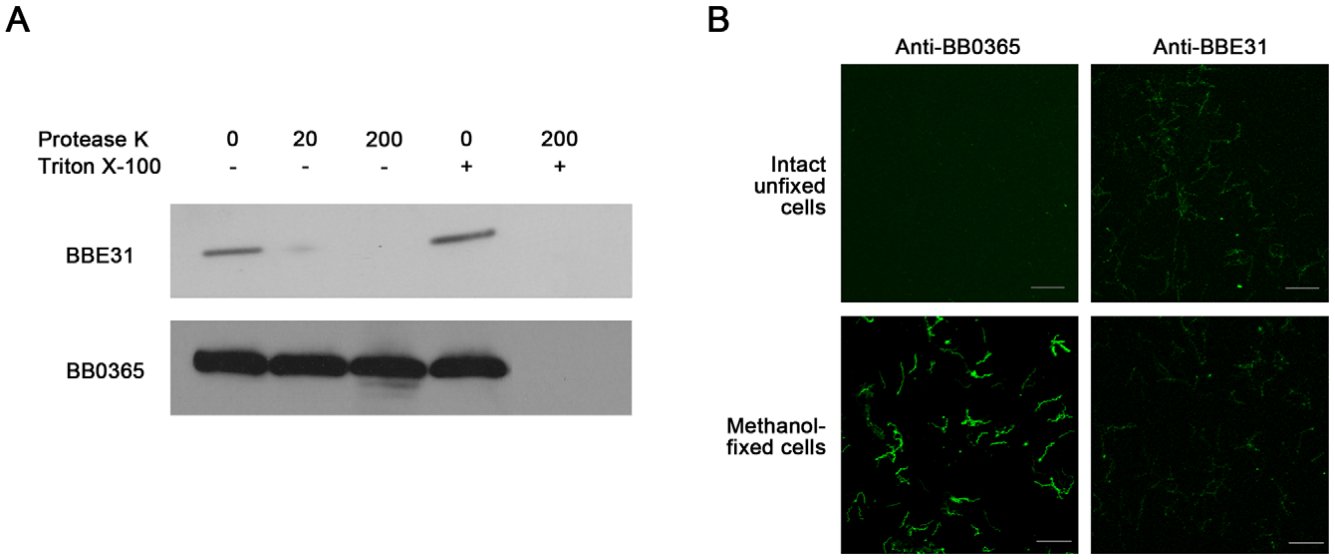
Subcellular localization of BBE31. (A) Protease digestion assay. Intact *B. burgdorferi* N40 cells were incubated with Protease K in the absence (-) or presence (+) of 0.05% Triton X-100. After digestion, cells were lysed and proteins were fractionated by SDS-PAGE. Immunoblots were developed with anti-BBE31 or anti-BB0365 (an inner membrane protein). 0, 20, 200: Protease K concentration is 0, 20 or 200 µg/ml. (B) Indirect immunofluorescence staining. Intact unfixed or methanol-fixed *B. burgdorferi* N40 were incubated with primary rabbit anti-BBE31 (right panels) or rabbit anti-BB0365 (left panels) antibodies, then incubated with the secondary antibodies Alexa 488-labelled goat anti-rabbit. Magnification, x63. Scale bar represents 20 µm.

Previous studies reported BBE31 as a *B.burgorferi* membrane protein [5]. To determine the exact localization of BBE31, both *in situ* proteolysis and indirect immunefluorescence assay (IFA) were carried out in this study. An inner membrane protein BB0365 was used as a negative control [15]. Surface-exposed proteins of intact bacteria can be degraded by proteases, whereas proteins not exposed to the surface are protected from proteolysis. In this work, when intact *B.burgdorferi* N40 were incubated with protease K, only BBE31 was degraded, while BB0365 was not (Figure 2A, lane 2 and lane 3). After disruption of *B.burgdorferi* cells by 0.05% Triton X-100, both proteins were completely degraded (Figure 2A, lane 5). These results indicate that BBE31 was degraded by protease K in the intact cells because of its outer-surface localization.

IFA was used to provide additional evidence of BBE31 localization. Initially intact unfixed or methanol-fixed *B.burgdorferi* N40 cells were incubated with rabbit anti-BBE31 or rabbit anti-BB0365 antibodies followed by detection with Alexa 488-labeled goat anti-rabbit secondary antibodies. We found that intact *B.burgdorferi* N40 cells can be labeled by anti-BBE31, but not anti-BB0365 (Figure 2B, row 1), while after disruption of the cellular outer membrane by methanol fixation, *B.burgdorferi* cells could be labeled by both antibodies (Figure 2B, row 2), further confirming that BBE31 is a *B.burgdorferi* outer-surface protein.

### BBE31 antiserum blocks *B.burgdorferi* migration from the *I.scapularis* gut to the salivary glands

Based on the *bbe31* expression profile - which was induced in the fed gut when compared to the salivary glands, and which was also not expressed in mice (Figure 1) - we predict that BBE31 may function during spirochete migration from tick gut to the salivary glands. To test this hypothesis, either 200 µl of normal rabbit serum or BBE31 antiserum was used to immunize C3H mice. 200 µl of BBI16 antiserum, another *B. burgdorferi* antigen, was used as the control. Twenty-four hours after the antisera injection, N40-infected nymphs were placed on mice. At 66-hour following the onset of feeding, ticks were removed from mice and dissected. Spirochete levels inside the tick gut and salivary glands were measured by both q-PCR and confocal microscopy.

**Figure 3:**
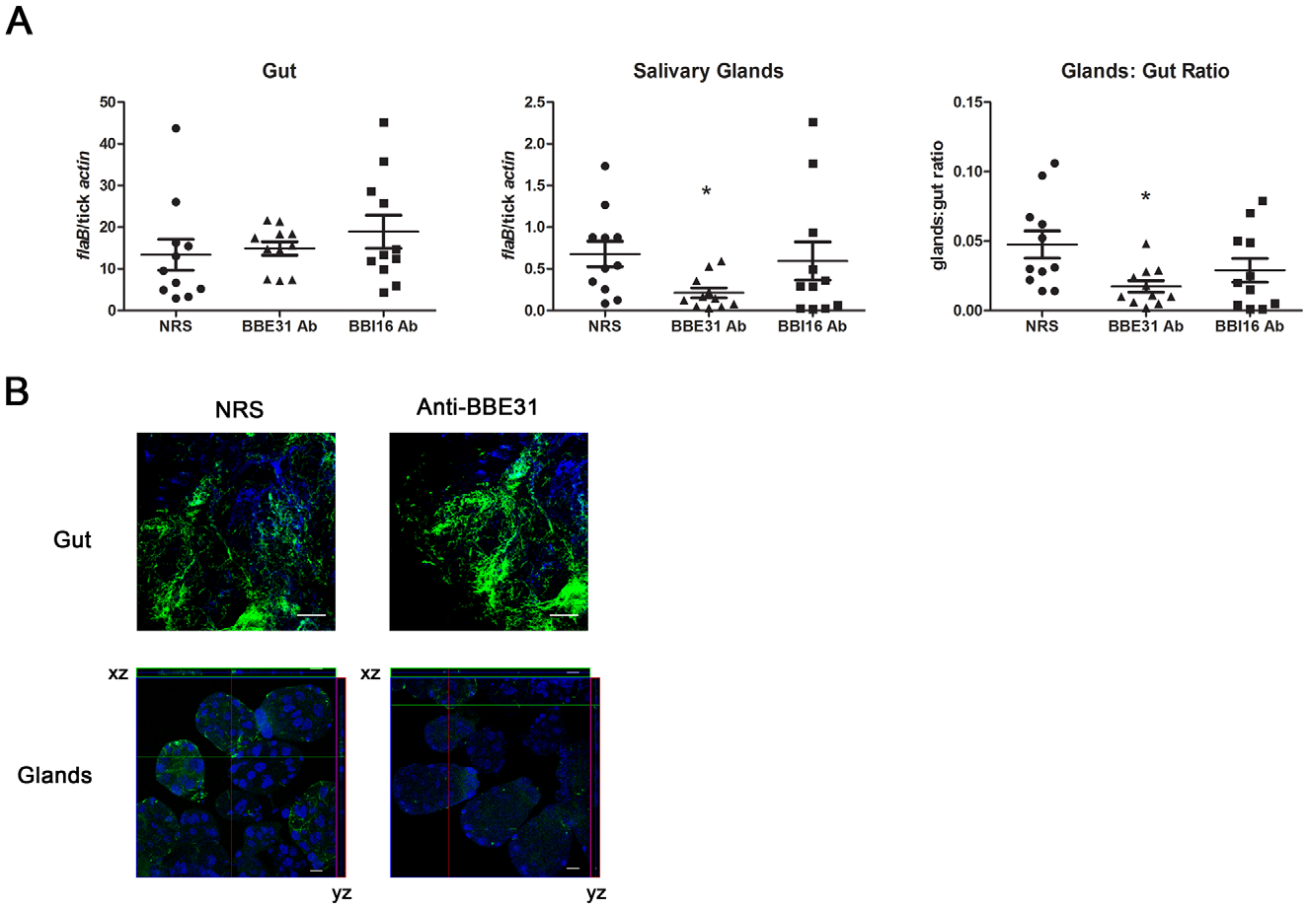
BBE31 antibodies block *B.burgdorferi* migration from *I.scapularis* gut to the salivary glands. (A) *B.burgdorferi* burden in *I.scapularis* gut and salivary glands assayed by q-PCR. N40-infected nymphs fed on C3H mice, which were injected with normal rabbit serum (NRS), BBE31 antiserum (BBE31 Ab) or BBI16 antiserum (BBI16 Ab), for 66 hours. DNA was extracted from both gut and salivary glands for q-PCR analysis. Each dot represents 5 guts or pairs of salivary glands. Data were collected from 3 studies of tick-feedings. *: P<0.05. (B) *B.burgdorferi* burden in the *I.scapularis* gut and salivary glands assayed by confocal microscopy. Murine immunization and tick feeding are the same as that in part (A). The spirochetes were probed with FITC-labeled goat anti-*borrelia* antibodies (shown in green), the nucleolus were stained with TO-PRO-3 (shown in blue). Orthogonal views (*xz* and *yz* axes) revealing the distribution of spirochetes through the full thickness of the infected salivary glands are shown. The gut and salivary gland samples were examined with the magnification of 63x and 25x, respectively. Scale bar represents 20 µm.

Q-PCR results revealed that both BBE31 and BBI16 antisera did not influence the *B.burgdoferi* burden in tick gut; however, BBE31 antiserum significantly decreased the spirochete burden in tick salivary glands (Figure 3A). Similar results were obtained from confocal microscopy. When compared to normal rabbit serum immunized mice, ticks fed on BBE31 antiserum-immunized mice showed lower *B.burgdoferi* burden in tick salivary glands (Figure 3B). These data indicated that BBE31 is important for *B.burgdoferi* migration from tick gut to the salivary glands during spirochete transmission from tick to the host.

Bactericidal effects of *B. burgdorferi* antibodies within ticks have been reported [16]. To make sure the decreased *B.burgdoferi* burden in tick salivary glands was not from the bactericidal effects of the antibodies, F(ab)_2_ fragments were prepared from either normal rabbit IgG or anti-BBE31 IgG, and were tested for borreliacidal activity. *B. burgdorferi* exposed to BBE31 F(ab)_2_ fragments showed spirochete count, motility and refractivity comparable to the control group that was grown in BSK medium without any treatment, indicating that BBE31 F(ab)_2_ fragments did not kill *Borrelia*.

To measure the influence of BBE31 F(ab)_2_ fragments on *B.burgdoferi* migration, mice were immunized with the F(ab)_2_ fragments as described [17]. Twenty-four hours after injection of F(ab)_2_ fragments, N40-infected nymphs were placed on mice and allowed to feed for 66 hours. The number of spirochetes inside the salivary glands or gut was also assessed by both q-PCR and confocal microscopy. Results were similar to those obtained in the antisera immunization experiments described above (Figure 3). BBE31 IgG F(ab)_2_ fragments decreased the *B.burgdorferi* burden in tick salivary glands but did not influence that in the gut (data not shown). Because the F(ab)_2_ fragments bind epitope(s) on BBE31 but have no bactericidal activity, these data indicate that BBE31 antibodies block *B.burgdorferi* migration by directly interfering with BBE31 function other than bactericidal effects.

### BBE31 antibodies partially protect mice from infection by tick transmitted *B. burgdorferi*

**Figure 4:**
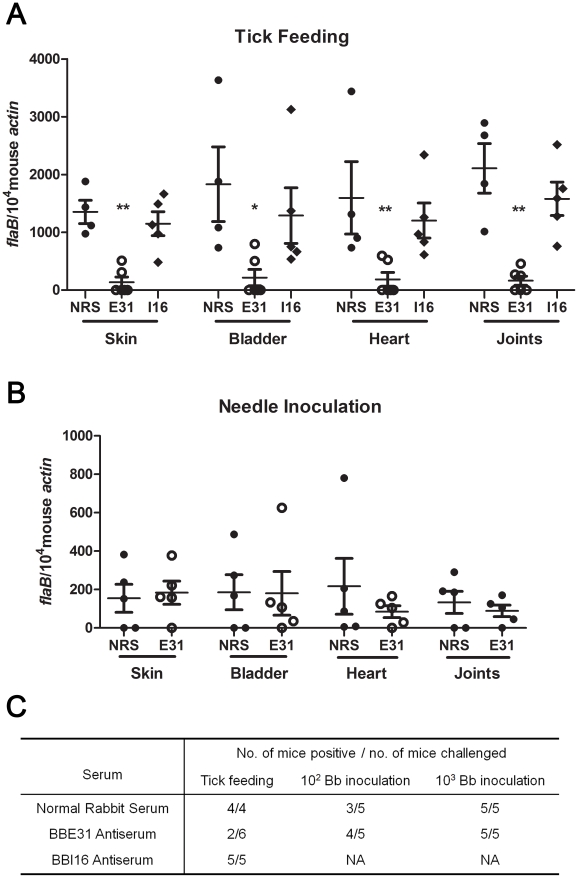
Influence of BBE31 antibodies on *B.burgdorferi* infectivity in mice. (A) BBE31 antibodies protect mouse from infection by tick-transmitted *B. burgdorferi*. Six of N40-infected nymphs were placed on 1 mouse, which has been injected with normal rabbit serum (NRS), BBE31 antiserum (E31) or BBI16 antiserum (I16), allowing to feed till exhausted. Twenty-one days later, the *B. burgdorferi* burden in mouse skin, bladder, heart and joints tissues were measured by q-PCR. Each dot represents 1 mouse. *: *p*<0.05, **: *p*<0.01. (B) BBE31 antibodies can't protect mice from infection by needle-inoculated *B. burgdorferi*. Mice were injected with rabbit normal serum (NRS) or BBE31 antiserum (E31). Twenty-four hours later, 10^2^ of cultured *B.burgdorferi* N40 were intraperitoneally inoculated into the mice. *B.burgdorferi* burden in murine tissues were measured 21 days after *Borrelia* inoculation. Each dot represents 1 mouse. (C) Mice protection by BBE31 antiserum determined by *in vitro* culture of skin, bladder, heart and joints. Mice tissues used for culture were the same as those described above (4A and 4B). 10^2^ Bb inoculation and 10^3^ Bb inoculation: 10^2^ or 10^3^ of *B.burgdorferi* N40 in BSK medium were needle-inoculated into each C3H mouse. NA: not assayed.

We then investigated whether the lower salivary glands infection consequently resulted in decreased mouse infection. N40-infected nymphs were placed on C3H mice injected with normal rabbit serum, BBE31 or BBI16 antiserum 24 hours prior to tick feeding. Ticks were allowed to feed to replete. Twenty-one days after tick feeding, mice were sacrificed and tissues including skin, heart, bladder and joints were cultured for spirochete growth. All mice treated with normal rabbit serum or BBI16 antiserum were infected. In contrast, only 2 of 6 mice treated with BBE31 antiserum were infected (Figure 4C). Q-PCR results showed that the infection level of mice treated by BBE31 antiserum was significantly lower than in animals treated by NRS or BBI16 antiserum (Figure 4A). This result suggests that BBE31 antiserum can partially protect mice from infection by tick-transmitted *B.burgdorferi*.

To test whether BBE31 antiserum protects mice from infection by needle-inoculated *B.burgdorferi* as well, 10^2^ or 10^3^ of N40 in BSK medium were inoculated intraperitoneally into C3H mice injected with 200 µl of BBE31 antiserum or normal rabbit serum. Twenty-one days after tick feeding, mice were sacrificed and tissues were cultured for spirochete growth. When the high dose was used, all the mice from both groups were infected. When the low dose was used, 3 of the 5 mice immunized with normal rabbit serum and 4 of the 5 mice immunized with BBE31 antiserum were infected (Figure 4C). Q-PCR results showed that both groups of mice were equally infected by *Borrelia* (Figure 4B). These results suggest that BBE31 antiserum can not protect mice from infection by needle-inoculated *Borrelia*, thereby confirming that BBE31 affects mouse infectivity through a tick transmission mechanism.

The needle-inoculation results are consistent with *bbe31* expression profile, which shows that the gene is expressed at very low level or not expressed *in vitro* or in mouse skin 1, 2 or 3 days after needle inoculation (Figure 1), indicating that BBE31 does not function in the early stage of host infection.

Two of the 6 mice immunized by BBE31 antiserum were infected by tick transmitted *Borrelia*, likely because a small proportion of *B.burgdorferi* invaded the salivary glands and were finally transmitted into the host (Figure 3B). Because BBE31 antiserum does not function in murine infection (Figure 4B), it is possible that these spirochetes established infection in the murine host. However, the infection levels were much lower than that of the NRS inmmunized mice (Figure 4A).

### BBE31 IgG F(ab)_2_ fragments block *B.burgdorferi* migration from tick gut into the hemolymph

When ticks take a bloodmeal, spirochetes replicate in tick gut and migrate through the hemolymph into the salivary glands. There are at least three steps involved in spirochete migration from gut to the salivary glands, penetration into hemolymph, survival in the hemolymphal environment and invasion of the salivary glands. Since we have shown that BBE31 antibodies can decrease spirochete burden in tick salivary glands, we next focused on investigating the exact step at which migration of spirochetes is inhibited.

**Figure 5:**
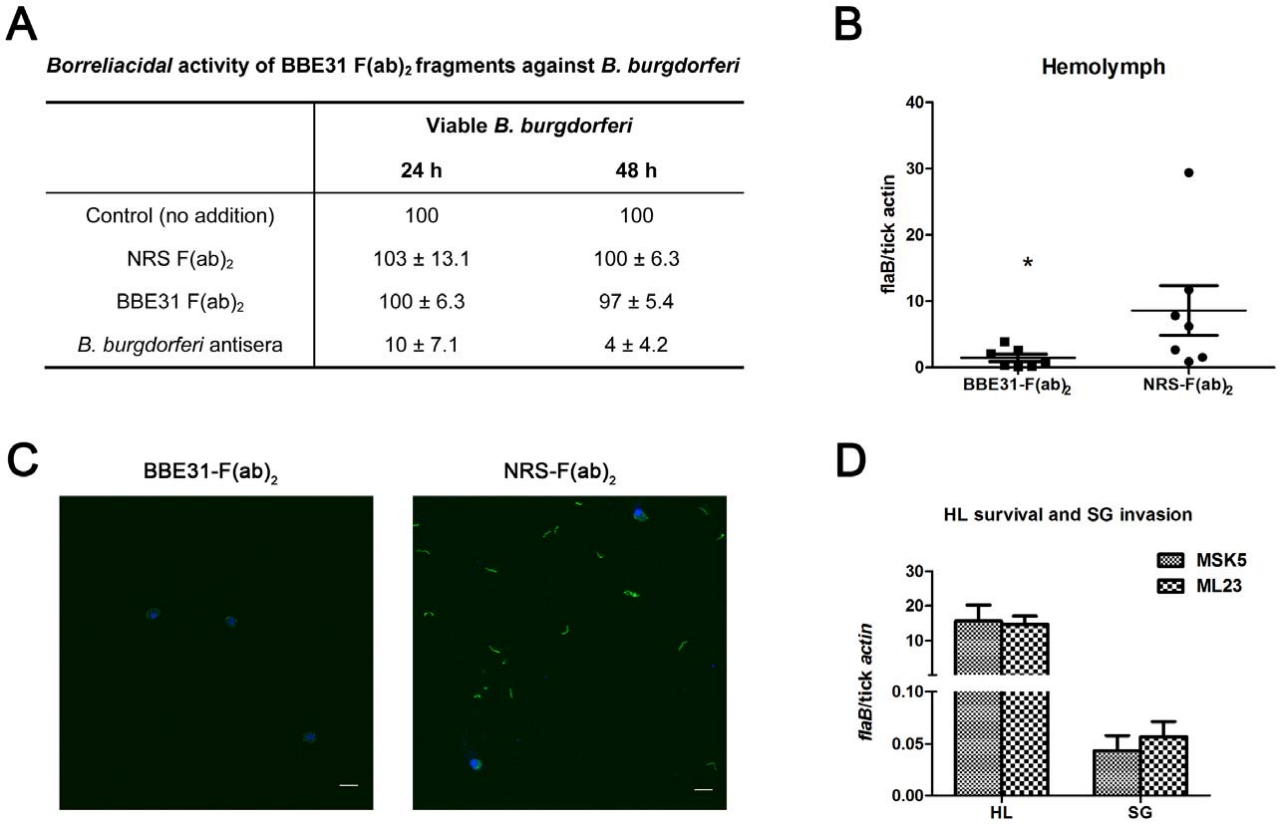
F(ab)_2_ fragments of anti-BBE31 IgG interfere with *B.burgdorferi* migration from *I.scapularis* gut into hemolymph. (A) *Borreliacidal* activity of BBE31 F(ab)_2_ fragments against *B. burgdorferi*. *B.burgdorferi* N40 were incubated in BSK medium in the absence (Control) or presence of F(ab)_2_ fragments prepared from normal rabbit serum [NRS F(ab)_2_] or BBE31 antiserum [BBE31 F(ab)_2_]. A borreliacidal serum from a patient with diagnosed Lyme Disease (*B.burgdorferi* antisera) was used as a positive control. The number of spirochetes was assessed by dark-field microscopy after 24 and 48 hours of incubation. Data are presented relative to controls without treatment. Data represent the number of spirochetes remaining viable after treatment (mean ± SD, n = 4). Difference between control and NRS F(ab)_2_- as well as BBE31 F(ab)_2_-treated samples were not statistically significant. (B) *B.burgdorferi* burden in *I.scapularis* hemolymph assayed by q-PCR. N40-infected nymphs fed on C3H mice, which had been injected with F(ab)_2_ fragments of either normal rabbit IgG (NRS-F(ab)_2_) or anti-BBE31 IgG (BBE31-F(ab)_2_), for 66 hours. DNA was extracted from the hemolymph for q-PCR analysis. Each dot represents hemolymph from 5 ticks. Data were collected from 2 times of tick-feedings. *: *p*<0.05. (C) *B.burgdorferi* burden in *I.scapularis* hemolymph assayed by confocal microscope. Tick feeding is the same as that described in part (A). The spirochetes in tick hemolymph were probed with FITC-labeled goat anti-*borrelia* antibodies (shown in green), the hemocyte nucleolus were stained with TO-PRO-3 (shown in blue). The FITC and TO-PRO-3 images were examined at ×25 magnifications and are presented as merged images. Scale bar represents 20 µm. (D) Spirochetes lacking *bbe31* can survive in tick hemolymph and invade the salivary glands. *B.burgdorferi* wild-type strain MSK5 and the lp25-lacking isolate ML23 were grown in BSK medium, resuspended in PBS and microinjected into partially-fed clean nymphal hemocoel (clean nymphs fed on C3H mice for 48 hours before microinjection). Six hours later, both hemolymph (HL) and salivary glands (SG) were collected for *B.burgdorferi* quantification by q-PCR. Mean and SD were calculated from 5 mRNA samples and 5 ticks were grouped for 1 mRNA sample.

We first tested whether BBE31 IgG F(ab)_2_ fragments influence *borrelial* burden in the tick hemolymph during tick feeding. N40-infected nymphs were allowed to feed on selected F(ab)_2_ fragments-treated mice for 66 hours. Hemolymph was collected as described in experimental procedures and the spirochete burden was measured by both q-PCR and confocal microscopy. When compared to ticks fed on mice treated with the normal rabbit IgG F(ab)_2_ fragments, ticks fed on BBE31 F(ab)_2_ fragments-treated mice showed a significantly lower spirochete burden in the hemolymph (Figure 5B & 5C). When we analyzed individual ticks, we found that the low hemolymph burden always correlated with decreased infection of salivary glands, suggesting that BBE31 antibodies decrease spirochete burden in tick salivary glands by reducing the spirochetes burden in the hemolymph and not by interfering with the spirochetes invasion of tick salivary glands.

Because *bbe31* is located in *B.burgdorferi* linear plasmid lp25, an lp25-missing strain ML23 was used to investigate whether BBE31 is necessary for the spirochete to survive in the hemolymphal environments. Both *B.burgdorferi* wild-type strain MSK5 and lp25-missing strain ML23 were grown in BSK medium, resuspended in PBS and microinjected into partially-feeding clean nymphal hemoceol (feed for 48 hours on clean C3H mice). Ticks were dissected 1 or 6 hours later. Q-PCR results indicated similar ML23 and MSK5 burden in tick hemolymph (Figure 5D, Left columns), suggesting that BBE31 is not necessary for *Borrelia* survival in tick hemolymph. Similarly, q-PCR results showed similar ML23 and MSK5 burden in tick salivary glands (Figure 5D, Right columns), further confirming that BBE31 is not important for *Borrelia* to invade tick salivary glands. Taken together, these results indicated that BBE31 play a role in *B.burgdorferi* migration from tick gut into the hemolymph.

### BBE31 binds to tick gut protein TRE31

**Figure 6:**
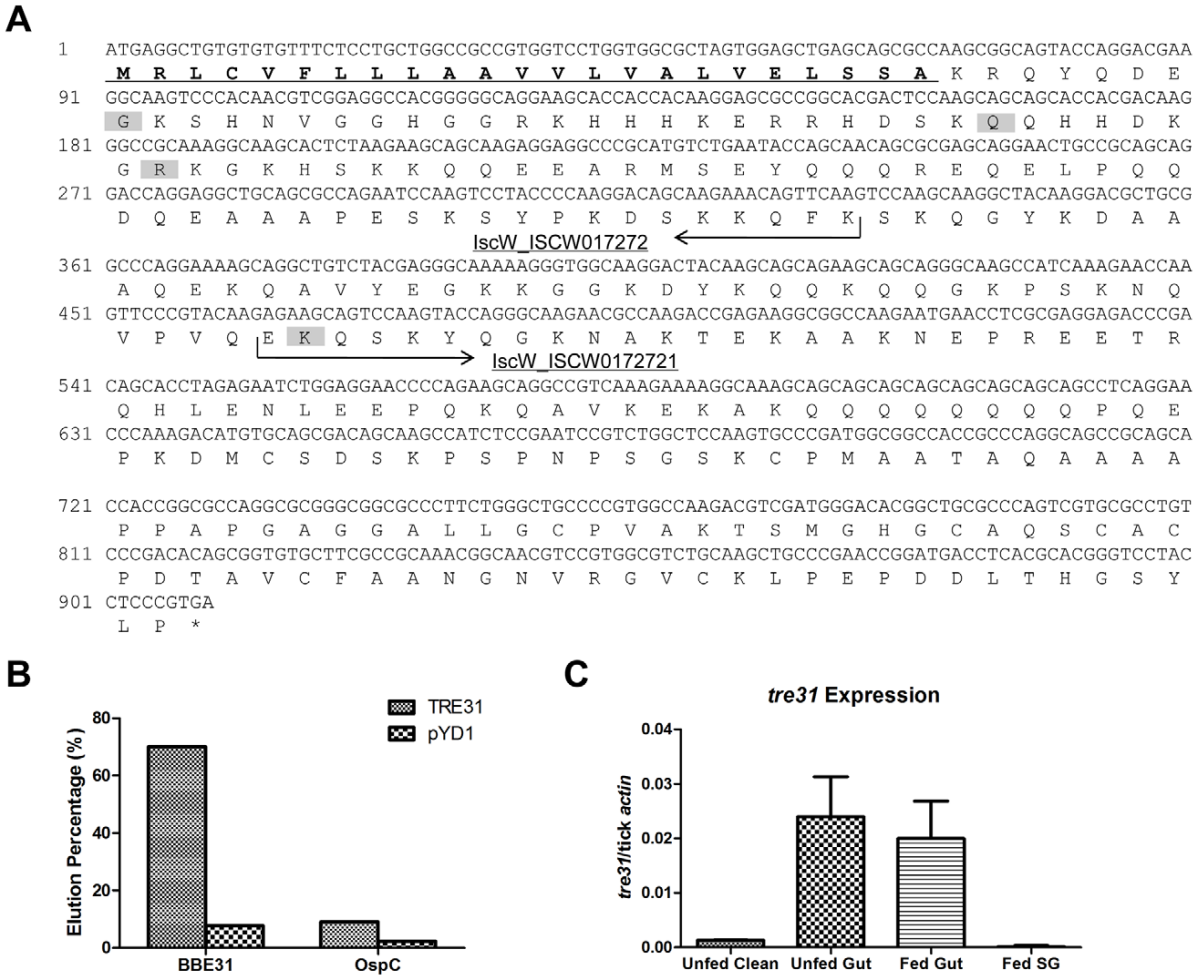
Confirmation of the interaction between BBE31 and TRE31 *in vitr*o. (A) Nucleotide and predicted amino acid sequences of TRE31. The *tre31* nucleotide sequence was deduced from the alignment of sequenced tick gut genes, which were expressed by yeast cells enriched through biotinylated BBE31, with several databases (BLAST search, GenBank and EMBL nonredundant databases). Two *I.scapularis* contigs IscW_ISCW017272 and IscW_ISCW017271 (arrows) were identified from the databases, which showed 100% and 98% nucleotide identity with *tre31* N-terminal and C-terminal sequences, respectively. The predicted peptide sequences of both contigs are in-frame with each other and constitute a protein of 302 amino acids. Among the 16 sequenced *tre31* copies expressed by enriched yeast cells, 4 different N-terminals were found (shading). The longest TRE31 copy is from amino acid 31 to 302. Protein sequence analysis through SignalP 3.0 revealed a signal peptide in the N-terminal (underlining) with the cleavage site between amino acid 23 (A) and 24 (K). (B) BBE31specifically interacts with yeast cells expressing TRE31. Plasmid containing *tre31* or the empty vector pYD1 was introduced into yeast competent cell EYB100, respectively. Both kinds of yeast cells were grown in SDCAA, induced in SGCAA, enriched through biotinylated BBE31 or OspC, and enriched cells eluted from the MACS column for quantification. Protein interaction was assessed by the elution percentage. (C) *tre31* expression in tick. *tre31* expression in unfed clean nymphal gut (Unfed Clean), N40-infected unfed nymphal gut (Unfed Gut), guts and salivary glands of N40-nymphs fed on C3H mice for 66 hours (Fed Gut and Fed SG), was measure by q-RT-PCR. Mean and SD were calculated from 6 mRNA samples.

Because BBE31 is outer-surface exposed, it is possible that BBE31 functions in ticks by interacting with specific tick protein. To investigate the mechanism(s) by which BBE31 facilitates *B.burgdoferi* migration from tick gut into the hemolymph, we screened a yeast surface-displayed tick-gut cDNA library for the identification of possible tick gut receptor(s) of BBE31. BBE31 was expressed in, and purified from *E. coli*, and labeled with biotin. Magnetic-activated cell sorting (MACS) was performed to enrich yeast clones expressing specific proteins interacting with the biotinylated BBE31. After four rounds of enrichment, plasmids were extracted from the enriched yeast cells and transformed into competent *E. coli* cells. DNA was isolated from Individual colonies and plasmid was sequenced. The experiments were repeated two times and a total of 30 clones were sequenced. Sixteen of the 30 clones expressed the same tick protein with similar C-terminal sequences. They were grouped into 4 different groups according to the initial amino acid of the N-terminal (Figure 6A, shading amino acids), indicating that at least 4 different copies of the gene exist in the cDNA library and were all bound by BBE31. The longest copy was then aligned with several databases (BLAST search, GenBank and EMBL nonredundant databases). We found that the N-terminal and C-terminal sequences showed significant similarity to *I. scapularis* contigs IscW_ISCW017272 and IscW_ISCW017271, respectively (Figure 6A). The predicted peptide sequences of both contigs were in-frame with each other and constituted a protein of 302 amino acids. Our longest copy corresponds to amino acids from 31 to 302 with protein sequence identity of 98%. This protein contains no putative domains and has no significant similarity to any other proteins outside *I.scapularis*. Because of its interaction with BBE31, we named it TRE31 (Tick Receptor of BBE31).

To retest the specific interaction between TRE31 and BBE31, the sequenced plasmid containing the *tre31* fragment from amino acid 31 to 312 (Figure 6A, predicted signal peptides removed) or empty vector (pYD1) was introduced into competent yeast cells (EBY100), respectively. 7.7% of yeast cells containing pYD1 were enriched by binding to biotinylated BBE31 while 70% of yeast cells containing *tre31* were enriched (Figure 6B). The 10-fold enrichment confirms a specific interaction between BBE31 and TRE31.

To test whether TRE31 specifically binds to BBE31 or could bind to other tick proteins as well nonspecifically, OspC was biotinylated and used to enrich the yeast cells expressing TRE31. Only 9.0% of yeast cells expressing TRE31 were enriched by biotinylated OspC (Figure 6B), thereby confirming the specificity of interaction between TRE31 and BBE31.

### TRE31 is predicted to be a secreted protein and specifically expressed in *B.burgdorferi*-infected tick gut

Because *B.burgdorferi* is an extracellular pathogen, TRE31 can interact with BBE31 *in vivo* only when it is secreted or outer-surface exposed. By SignalP 3.0 analysis [18], a signal peptide was found in the N-terminal of TRE31 with a cleavage site between amino acid 23 and 24 (Figure 6A). The signal peptide enhances the possibility that TRE31 is secreted.

In order to interact with each other, interacting partners should be expressed within a similar time frame. Upon measurement of *tre31* expression in different tick tissues, including unfed clean-tick gut, unfed N40-tick gut, fed gut and fed tick salivary glands, *tre31* was found to be induced by *B.burgdorferi* infection, and expressed in both unfed and fed gut, but not expressed in tick salivary glands (Figure 6C). Our previous results have shown that *bbe31* has the highest expression in the fed tick gut (Figure 1). Co-expression of TRE31 and BBE31 in the fed gut suggests an *in vivo* interaction of the two proteins.

### *tre31*-deficient ticks have reduced *B.burgdorferi* burden in the hemolymph and salivary glands

**Figure 7:**
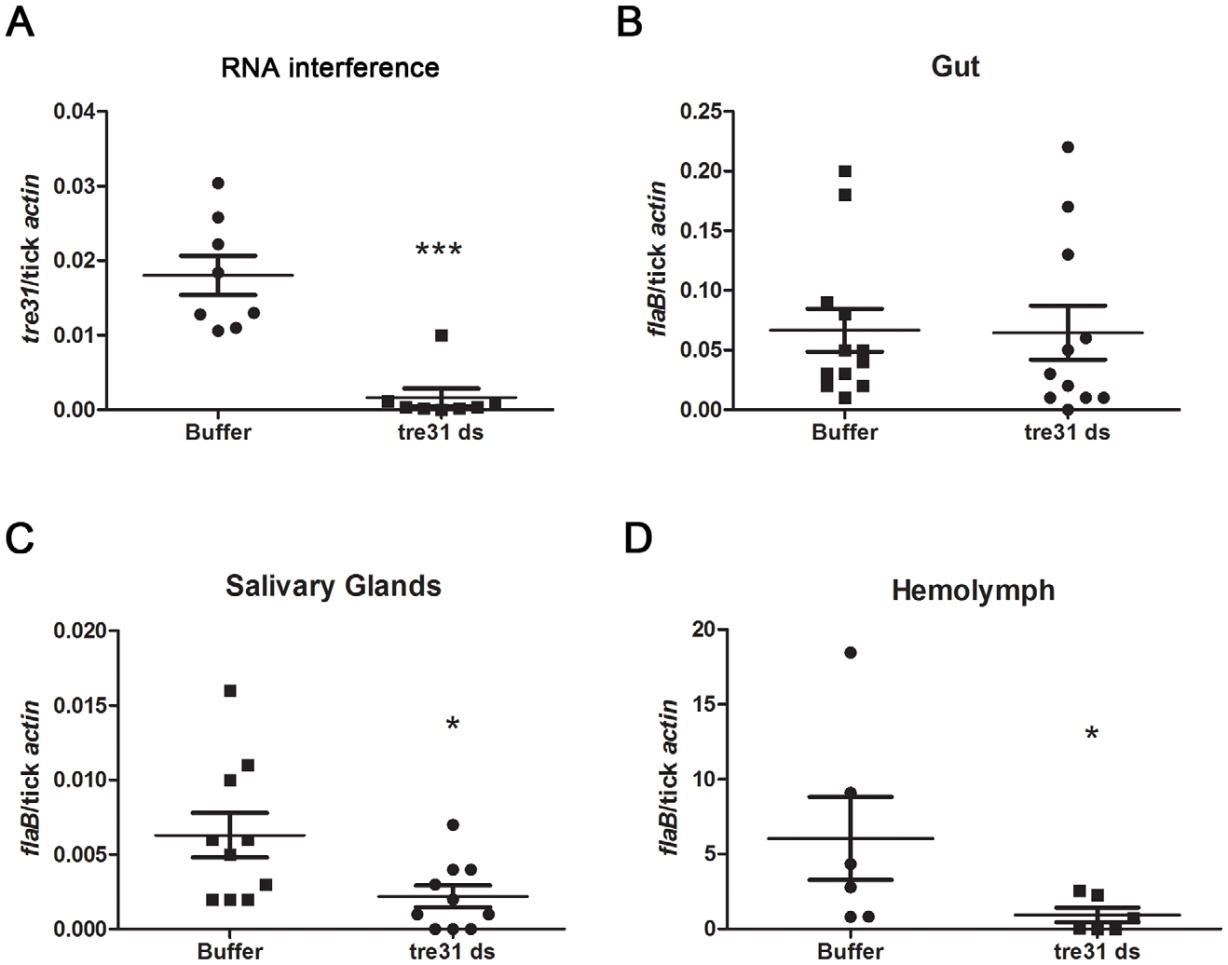
*tre31 ds*RNA interfered with the migration of *B.burgdorferi* within *I.scapularis* during spirochete transmission from tick to the host. (A) *tre31* mRNA was dramatically reduced within *tre31 ds*RNA-treated tick guts. Equal volumes of *tre31 ds*RNA (tre31 ds) or Elute Buffer (Buffer) were delivered directly into the gut of N40-infected *Ixodes* nymphs via microinjection. Ticks were allowed to rest for 3 hours and feed on C3H mice for 66 hr. *tre31* expression in tick gut was measured by q-RT-PCR. (**: *p*<0.01) (B) *B.burgdoferi* burden in the tick gut was not influenced by *tre31* deficiency. (C) *B.burgdoferi* burden in tick salivary glands was decreased by *tre31* deficiency (*: *p*<0.05). (D) *B.burgdoferi* burden in tick hemolymph was decreased by *tre31* deficiency (*: *p*<0.05). Each dot represents 2 tick gut or salivary glands, or 4 hemolymph. Experiments were repeated two times.

To determine the role of TRE31 in mediating *B.burgdorferi* migration within ticks, we generated *tre31*-deficient nymphal ticks using RNA interference (RNAi). Equal volumes of *ds*RNA or elute buffer were delivered directly into the gut of N40-infected *Ixodes* nymphs via microinjection. Ticks were allowed to rest for 3 hours and placed on C3H mice. At 66-hrs following the onset of feeding, ticks were dissected and q-RT-PCR analysis indicated a dramatic reduction in the level of *tre31* mRNA within *tre31 ds*RNA-treated tick guts when compared to the control group (Figure 7A).

We then assessed whether migration of spirochetes was affected by RNAi-mediated *tre31* deficiency. *B.burgdorferi* burden in tick gut, salivary glands and hemolymph were assessed through q-RT-PCR. Compared to the control group, *tre31 ds*RNA-treated ticks showed similar *B.burgdorferi* numbers in the tick gut (Figure 7B) and significantly lower spirochete numbers in tick hemolymph (Figure 7D, P<0.05) and salivary glands (Figure 7C, P<0.05), indicating TRE31 functions in enabling migration of spirochetes within the vector during spirochete transmission from tick to the host.

## Discussion

*B. burgdorferi* tick-to-host transmission is a complex process. At least 6 steps are involved in the transmission. When tick feeding commences, *B. burgdorferi* rapidly multiply in the feeding gut, some of the spirochetes dissociate from the gut, penetrate into hemolymph, combat the hemolymphal environment, invade salivary glands and finally are transmitted into the host via expelled saliva [9], [19], [20]. Several *B. burgdorferi* lipoproteins are necessary for spirochete tick-to-host transmission; however, because these proteins were identified using whole ticks as the template, no other protein except for OspA and OspC have been linked to a specific step [7], [10], [11], [17], [21]. In this study, the BBE31 function in the tick was revealed in detail and the mechanism of action was investigated. We demonstrated that BBE31 is required for spirochete migration from tick gut into the hemolymph. A tick protein TRE31 enables *Borrelial* movement by interacting with BBE31. The decreased hemolymph infection results in lower salivary glands infection, which eventually attenuates mouse infection by tick-transmitted *B.burgdorferi*.

Several lines of evidences demonstrate that BBE31 functions in *B.burgdorferi* penetration from the fed gut into the hemolymph. Firstly, BBE31 IgG F(ab)_2_ fragments do not influence the *B.burgdorferi* burden in the tick gut, while they decrease the bacterial burden in both hemolymph and salivary glands. When we analyzed individual ticks fed on BBE31 antibodies-treated mouse, we found lower tick salivary glands infection always correlated to lower hemolymph infection, suggesting that the decreased *B.burgdorferi* burden in tick hemolymph is the reason for the lower bacterial burden in salivary glands.

Decreased *B.burgdorferi* burden in the tick hemolymph could be either due to less spirochete penetration into the hemolymph or that the penetrated spirochetes are killed in the hemolymph. *I. scapularis* has been revealed to be immunotolerant to *B. burgdorferi* challenge, the spirochetes are not killed within at least 1 hour of infection which is enough time for the spirochetes to invade the salivary glands [20]. Specific *B. burgdorferi* factors might be necessary to combat the phagocytic response spirochetes encounter in tick haemocoel [22]. If BBE31 is necessary for *Borrelia* to survive in the hemolymphal environment, a *bbe31*-deficient strain will be easily cleared from the hemolymph. In this study, we found that the *bbe31*-deficiency strain ML23, when compared to the wild-type strain MSK5, showed a similar ability to live in the *I.scapularis* hemocoel after 1 or 6 hours of microinjection, indicating that at least the gene itself is not necessary for *B.burgdorferi* survival in the *I.scapularis* hemocoel. We have also tried to generate *B. burgdorferi* that specificially lack *bbe31*. While we have been able to generate numerous other knockout spirochetes [17], [23], [24], [25], [26], we have not been able to obtain *bbe31*-deficient *B. burgdorferi*, despite repeated attempts.

*Borrelia* migration from tick gut into the hemolymph is a complex process. A recent research delineated the routes and behavioral patterns that *Borrelia* utilize to disseminate within the *Ixodes* ticks by imaging a live, infectious *B. burgdorferi* strain expressing GFP [19]. At the onset of feeding, the replicating spirochetes form networks of nonmotile organisms that advance toward the basolateral surface of the epithelium while adhering to epithelial cells. The nonmotile spirochetes then transit into motile organisms that penetrate the basement membrane and enter the hemocoel [19]. It is imaginable that this complex migration requires diverse interactions between *Borrelia* outer-surface proteins and vector ligands. The identification of new *borrelia*-tick interactions will enhance our understanding of *Borrelia* migration. In this study, we have demonstrated that both BBE31 antibodies and *tre31*-dificiency decreased the numbers of *Borrelia* entered hemolymph; however, it remains unclear that how the BBE31-TRE31 interaction helps the bacteria to move within or escape from the fed gut.

*bbe31* is present on *B.burgdorferi* plasmid lp25 which has been shown to be necessary for both mouse and tick infection[27], [28], [29]. Two genes locate in lp25 have been reported to be important for animal and/or vector infection. BBE22, which encodes a nicotinamidase, has been revealed to replace the requirement for lp25 during mammalian infection [30]. BBE16 is essential for the spirochete's persistence in tick. Along with BBE22, it is also required for achieving natural levels of *Borrelia* infectivity in mice [31]. Because the *B.burgdorferi* strain missing lp25 does not efficiently infect the tick gut, no work has focused on function of lp25 in *B.burgdorferi* gut-to-salivary glands migration. Moreover, it remains possible that other lp25-encoding genes work in concert with BBE16 for maximal infectivity of *Borrelia* in its vector. In this study, in addition to determining the functions of BBE31 in *B.burgdorferi* gut-to-salivary glands migration, we also investigated whether BBE31 plays a role in larva or nymph infection and in the bacterial persistence through the molting periods. When BBE31 antiserum or normal rabbit serum was used to immunize *B.burgdorferi*-infected C3H mice, clean larvae or nymphs fed on both groups of mice could be equally infected by *B.burgdorferi*. The acquired *B.burgdorferi* could persist through the molting period, both from larva to nymph and from nymph to adult. These data suggest that BBE31 is not important for *B.burgdorferi* acquisition and persistence (data not shown).

In summary, for the first time, a *borrelial* lipoprotein BBE31 has been linked to the spirochete gut-to-hemolymph movement, and the vector ligand of BBE31 has been characterized. Understanding how microbes migrate through the hemolymph of their corresponding arthropod vectors is poorly understood, and we hope that this study will serve as a paradigm for other infectious diseases of medical importance. A detailed knowledge of these specific types of pathogen-vector interactions may lead to development of novel vaccine strategies that can be rationally designed to target the microbial life cycle within the vector.

## Materials and Methods

### *Borrelia*, ticks and mice

A low-passage clonal isolate of *B. burgdorferi* N40 that is infectious for mice was used throughout the study except for the experiments involving lp25-deficient *B. burgdorferi*. Lp25-missing strain ML23 is derived from a clonal, low-passage, virulent *B. burgdorferi* strain MSK5. N40-infected nymphs were produced in our lab by clean larval *I. scapularis* feeding on N40-infected C3H/HeN mice. Female C3H/HeJ (C3H) mice, 5 weeks of age, were obtained from the Jackson Laboratory.

### Ethics statement

Animals were housed and handled under the Guide for the Care and Use of Laboratory Animals of the National Institutes of Health. The animal experimental protocol was approved by the Yale University's Institutional Animal Care & Use Committee (Protocol Permit Number: 2008-07941). All animal infection experiments were performed in a Bio-safety Level 2 animal facility, according to the regulations of Yale University.

### Tissues collection from fed ticks

Fed ticks that detached from mice were dissected. Forelegs were severed at the cocal-thochanteral joint and hemolymph was drawn to the tip of a clean blade. Ticks were then dissected in PBS both the gut and salivary glands were isolated. Salivary glands were washed twice in PBS for removal of any contaminating free spirochetes.

### *bbe31* expression throughout *B.burgdorferi* life cycle

For quantitative analysis of *bbe31* expression *in vitro*, *B.burgdorferi* N40 were grown in BSK medium to a concentration of 1×10^7^/ml before total RNA was extracted. For *bbe31* expression during larva acquisition and persistence through the inter-molt stage, C3H mice were inoculated with 1*10^6^ of *B.burgdorferi* N40. Fourteen days later, murine infection was assessed and every 50 larvae were allowed to feed on each infected mouse till engorged. Total RNA was extracted from the whole body of larva immediately, 4- or 21-day after larva feeding. For *bbe31* expression during *B.burgdorferi* tick-to-host transmission, 15 N40-infected nymphs were allowed to feed on each C3H mouse. Ticks were detached from the mouse at 24, 48 or 72-hour following the onset of feeding. For *bbe31* expression during the first 3 days of murine infection, 1*10^6^ *B.burgdorferi* N40 in 200 µl of BSK medium were inoculated into C3H mouse near the ear area. Ear patch was taken 24, 48 or 72 hours after inoculation. For *bbe31* expression in specific tissues of infected mice, every 10 of N40-infected *I.scapularis* nymphs were allowed to feed on each mouse till engorged. Twenty-one days later, mice were sacrificed and total RNA was extracted from the infected tissues. RT-PCR and SYBR-Green based q-PCR were performed according to the protocols provided by the manufactures. *flaB* was amplified as an internal control for the loading of cDNA isolated from different samples. H_2_O was used as a negative control to exclude any non-specific amplification.

### Antibodies and the generation of F(ab)_2_ fragments

For generation of BBE31 or BBI16 polyclonal antibodies against *B. burgdorferi* N40, both genes without the signal peptide sequences were amplified from N40 genomic DNA and were cloned into the expression vector pGEX-6P-2 through restriction sites of *BamHI* and *NotI* (Primers listed in Table S1). The recombinant plasmids were transformed into the BL21-CodonPlus-RIL competent cells (Stratagene Cat#230240). The target proteins were expressed with N-terminal GST-tag which can be removed by the PreScission protease (GE Healthcare #27-0843-01). Purified proteins were used to immunize rabbits to produce rabbit anti-BBE31 or anti-BBI16 antisera. The F(ab)_2_ fragments were prepared from normal rabbit serum or BBE31 antiserum as described [17]. Briefly, IgG was purified from 10 ml of normal rabbit serum or polyclonal antiserum by the sera passed over a 0.5-ml protein G sepharose-column (GE Healthcare Cat#17-0618-01). The eluted IgG was dialyzed overnight against 20 mM sodium acetate buffer at pH 4.5 (Tube-O-Dialyzer 4 kDa Midi, G-Biosciences Cat#786-616) and was concentrated to 8 mg/ml. F(ab)_2_ fragments were generated from the whole IgG using the Pierce F(ab')_2_ Preparation Kit (Pierce Cat#44988) according to the protocols provided by the manufacturer.

### Bactericidal assay

F(ab)_2_ fragments were tested for their bactericidal activity against *B. burgdorferi* N40 by dark-field microscopy as described previously [32]. Briefly, spirochetes (5×10^6^/ml) were incubated in BSK medium supplemented with purified F(ab)_2_ fragments (50 µg/ml) for 48 hours at 33°C. Anti–*B. burgdorferi* serum from a patient with diagnosed Lyme arthritis served as a control in the bactericidal assay. The percentage of viable spirochetes was determined by dark-field microscopic observation of the loss of spirochete motility and refractivity. In addition, 50-µl aliquots from each tested sample were incubated with 500 µl of BSK medium at 33°C for 5 days. The *B. burgdorferi* cells were then counted, and these results were compared with the initial viability by dark-field microscopy.

### Q-PCR and confocal microscopy to assess *B.burgdorferi* migration within *I.scapularis* nymphs

Clean C3H mice were passively immunized with 200 µl of BBE31 antiserum, BBI16 antiserum (negative control) or normal rabbit serum (negative control), respectively. Ten *B. burgdorferi-*infected nymphs were placed on each mouse 24 hours after immunization. At 66-hour following the onset of feeding, ticks were dissected and the *B.burgdorferi* burden in the collected gut, salivary glands and hemolymph was analyzed by both q-PCR and confocal microscopy.

To investigate the influence of F(ab)_2_ fragments on spirochete migration, clean C3H mice were injected as described [17]. Briefly, the F(ab)_2_ fragments were diluted in PBS (pH 7.4) to 50 µg/ml. Mice were injected with selected F(ab)_2_ fragments (100 µl intraperitoneally and 100 µl subcutaneously). 10 *B.burgdorferi-*infected nymphs were placed on each mouse 24 hours after immunization. Mice were treated again with F(ab)_2_ fragments on the next day to maintain an effective concentration of F(ab)_2_ in the blood. Nymphs were removed at 66-hour following the feeding commence and pathogen burden were analyzed by q-PCR and confocal microscopy.

For q-PCR analysis, DNA was extracted from gut, salivary glands and hemolymph. Pathogen burden in different tick tissues was determined by measuring *flaB* copies using SYBR-Green based-qPCR. *I.scapularis ß-actin* (primers listed in Table S1) was amplified as an internal control for the DNA loading.

For confocal microscopy analysis, gut, hemolymph or salivary glands were placed on silylated glass slides (Sigma Cat#S4651) and allowed to dry. Guts were fixed with ice-cold acetone for 5 minutes; salivary glands were fixed in 4% PFA at room temperature for 30 minutes; while hemolymph does not need to be fixed. The slides were rinsed twice with PBS. For guts and salivary glands, the slides were incubated in PBS +2% Tween 20+2% FBS for 30 minutes and then incubated with 100 µl of 1∶50 diluted FITC-labeled goat anti-*B.burgdorferi* (KPL Cat#02-97-91) at room temperature for 1 hour. The slides were then rinsed three times with PBST and the nucleolus was stained with TO-PRO-3 iodide (Invitrogen Cat#T3605) at room temperature for 3 minutes. Same protocols were used for hemolymph, except that PBST replaced by PBS. The samples were examined with a Zeiss LSM 510 confocal microscope.

### Murine infection via tick transmission

Clean C3H mice were passively immunized with 200 µl of BBE31 antiserum, BBI16 antiserum (negative control) or normal rabbit serum (negative control), respectively. Twenty-four hours after immunization, six *B. burgdorferi*-infected nymphal ticks were placed on each mouse. Twenty-one days after tick repletion, mice were sacrificed and examined for *Borrelia* infection by *in vitro* culture of the bladder, heart, skin, and joints tissues. In addition, the tissues were examined by q-PCR for spirochetes burden using *flaB* primers. The murine *ß-actin* gene (primers listed in Table S1) was amplified as an internal control for the DNA loading.

### Murine infection via needle inoculation

Clean C3H mice were passively immunized with 200 µl of BBE31 antiserum or normal rabbit serum (negative control). Twenty-four hours after immunization, mice were challenged with an intraperitoneally inoculation of 10^2^ or 10^3^ of *B.burgdorferi* N40. Murine infection by spirochetes were examined as described above.

### Proteolytic sensitivity of BBE31

*B.burgdorferi* N40 cultured in BSK medium was harvested in the mid-log-phase, resuspended in PBS at a cell density of 1*10^7^/ml, and equally split into 5 tubes. The aliquots were treated with 20 or 200 µg of Protease K, 0.05% Triton X-100, 200 µg of Protease K with 0.05% Triton X-100 or remained not to be treated. After 1 hour of incubation at room temperature, cells were lysed and proteins were fractionated by SDS-PAGE. Western blotting was then performed with anti-BBE31 or anti-BB0365 sera (negative control), respectively.

### Indirect immunofluorescence of *B. burgdorferi*

Intact *B.burgdorferi* N40 cells suspended in PBS were attached to silylated glass slides. For each analyzed protein, two slides were prepared. After air drying, one slide was fixed by immersion in 100% methanol for 10 minutes and the other remained not to be fixed. Both kinds of slides were rinsed twice with PBS. Then the spirochetes were incubated with rabbit anti-BBE31 or rabbit anti-BB0365 sera (negative control), which had been diluted in PBS +2% FBS, for 1 hour. The slides were rinsed again with PBS, and the spirochetes were incubated with Alexa 488-conjugated goat anti-rabbit IgG (in PBS +2% FBS) for 1 more hour. After three rinses with PBS, the slides were dried and observed with a Zeiss LSM 510 confocal microscope.

### *B.burgdorferi* survival in the hemolymph and invasion of salivary glands

*B.burgdorferi* strain MSK5 or ML23 (lp25-missing) were grown in BSK medium to mid-log phase, harvested and resuspended in PBS at a cell density of 1*10^7^/ml. 50 nl of *B.burgdorferi* MSK5 or ML23 was microinjected into the hemocoel cavity of clean nymphs, which has been fed on clean mouse for 48 hours. Ticks were allowed to stay at room temperature for 1 or 6 hours before dissection. The *B.burgdorferi* burden in either hemolymph or salivary glands was determined by measuring *flaB* copies using q-PCR.

### Yeast surface display to identify tick gut protein interacting with BBE31

cDNA was prepared from the guts of clean *I.scapularis* nymphs which have been fed on clean C3H mice for 72 hours before dissection. The cDNAs were cloned into pYD1 yeast display vector (Invitrogen Cat#V835-01). Genes were expressed as a fusion protein with the yeast agglutinin protein Aga2p, can be secreted and displayed on yeast surface. As a quality control of the generated tick gut-cDNA library, we checked the expression of several representative tick genes including *β-actin*, *salp25D* and *trospA*, and found that all the three genes could be amplified from the yeast library (Data not shown) [10], [33]. Yeast cells expressing tick protein interacting with BBE31 was isolated according to the protocols described [34] with several modifications. All the mediums and buffers are the same as that described [34]. Yeast cells were always incubated at 30°C and 250 r.p.m., and centrifuged at 2,500 g for 5 minutes. BBE31 was labeled with biotin according to the protocols (Invitrogen Cat#6347).

1×10^9^ yeast cells were thaw at room temperature and grow overnight in SDCAA medium to an absorbance of 6–8 at 600 nm. Pellet the yeast cells and wash twice with sterile water to remove any dextrose. The yeast cells were then inoculated into SGCAA medium at OD_600_ of 0.5, grown overnight to induce the library expression. Pellet 3*10^8^ of yeast cells and resuspend in 1 ml of PBSM buffer. 30 µg of biotinylated BBE31 were added into the yeast cell suspension, incubating at 4°C for 1 hour. The yeast cells were pelleted again and resuspended in 1 ml of PBSM buffer. 100 µl of streptavidin microbeads (Mitenyi Biotec Cat#130-048-101) were added into the suspension, incubating at room temperature for 30 min. For MACS separation, place an LS column (Mitenyi Biotec Cat# 130-042-401) onto the magnet and stand assembly. Wash the column with 5 ml PBSM buffer to equilibrate. Yeast cells from last step were applied to the column. When cells have passed through the column, wash the column with 10 ml of PBSM buffer. To elute cells, remove column and add 5 ml SDCAA medium to the column and use the plunger supplied with the column to push the remaining cells through. Repeat to elute two more times and collect all the eluted cells. Add SDCAA medium to a final volume of 50 ml, add pen-strep solution (1∶100 dilution) and propagate eluted yeast for subsequent round of sorting. After 4 rounds of sorting, plasmids were isolated from the enriched yeast cells using a Zymoprep kit according to the manufacture's instruction (Zymo Research Cat#D2004). Transform 2 µl of plasmid DNA into *E.coli* TOP10 competent cells. Plasmids were isolated from single *E.coli* clone for sequencing with pYD1 forward primer: 5′-AGTAACGTTTGTCAGTAATTGC-3′ and pYD1 reverse primer: 5′-GTCGATTTTGTTACATCTACAC-3′.

To confirm the interaction between tick protein TRE31 and BBE31, plasmids containing sequenced *tre31* were transformed into the yeast competent cells EBY100. The empty plasmid pYD1 was also transformed into EYB100 as negative control. Both kinds of yeast cells were grown in SDCAA and induced in SGCAA. 3*10^8^ of each kind of yeast cells were used to sort by biotinylated BBE31 according to the protocols described above. Yeast cells bound to BBE31 were eluted with 10 ml of SDCAA. OD_600_ was measured and the elution percentage was calculated to estimate the interaction between TRE31 and BBE31. To test whether TRE31 bind to other *B.burgdorferi* lipoprotein like OspC, biotinylated OspC replacing BBE31 was used in the above experiments.

### RNA interference

A 570-bp *tre31* fragment was PCR amplified using the primers tre31-si-F and tre31-si-R (Table S1). *ds*RNA was synthesized and purified using a commercial kit (Megascript RNAi Kit, Ambion Cat#AM1626). For *tre31* silencing in the tick gut, 20 nl of the *tre31 ds*RNA (1 µg/µl) or buffer (as control) were microinjected into the gut of unfed N40-nymph as described, respectively (Pal et al., 2004). Ticks were allowed to rest for 3 hours and placed onto C3H mice. At 66-hour following onset of feeding, the nymphs were detached from the mice. RNA was extracted from tick gut to assess *tre31* silencing and *B. burgdorferi* burden, from salivary glands and hemolymph to assess *B. burgdorferi* burden. q-PCR primers for assessing *tre31* silence efficiency are tre31-QF and tre31-QR (Table S1).

### Accession numbers

The GenBank accession numbers for genes and proteins related with this study: BBE31/NP_045436; BBI16/NP_045547; TRE31 was sequenced in this study and submitted to GenBank, the accession number is HQ998856.

## Supporting Information

Table S1 Primers used in this study.
(DOC)
