# Supplementary material for: Molecular Interactions that Enable Movement of the Lyme Disease Agent from the Tick Gut into the Hemolymph
Source: PLoS Pathog. 2011 Jun 9;7(6):e1002079. doi: 10.1371/journal.ppat.1002079 (PMC3111543; doi:10.1371/journal.ppat.1002079)
Supplement: Table S1 — Primers used in this study. (DOC) [file ppat.1002079.s001.doc]

**Table S1. Primers used in this study.**

| **Primers** | **Sequences (5’-3’)** | **Purpose** |
| --- | --- | --- |
| bbe31-F | TGGAAGAACTTTTGATACAGCGAT | q-PCR primers for *bbe31* |
| bbe31-R | TTTCAAACGAATTTTTAAGCTTCTCT |
| bbi16-F | AAGCCCCCTGGATCCTCA | q-PCR primers for *bbi16* |
| bbi16-R | TCAAGAGCTCCTCCAATAGCG |
| flaB-F | TCAAGCGTCTTGGACTTTAAGAGTT | q-PCR primers for *flaB* |
| flaB-R | CACCAGAGAAAAGATTTGCAACATTA |
| T-actin-F | GGCGACGTAGCAG | q-PCR primers for *I. scapularis ß-actin* |
| T-actin-R | GGTATCGTGCTCGACTC |
| M-actin-F | AGCGGGAAATCGTGCGTG | q-PCR primers for mouse *ß-actin* |
| M-actin-R | CAGGTACATGGTGGTGCC |
| Ex-e31-F | GGATCCATGAATCCAGATTCTAATACCAATCAA | Amplify *bbe31* for protein expression in *E.coli* |
| Ex-e31-R | GCGGCCGCTTAAAGGTTGCTTATTGACAATATG |
| Ex-i16-F | CCCGGGATGAGGCCGGATTTTAATATCG | Amplify *bbi16* for protein expression in *E.coli* |
| Ex-i16-R | GCGGCCGCTTAAAGTTTATATTTTGACACTATAAGC |
| tre31-QF | CAGCACCACGACAAGGGC | q-PCR primers for *tre31* |
| tre31-QR | TGCGGCAGTTCCTGCTC |
| tre31-si-F | TAATACGACTCACTATAGGGAGAGAGGCCCGCATGTCTGAATA | Amplify *tre31* for RNA silence |
| tre31-si-R | TAATACGACTCACTATAGGGAGACGTGTCCCATCGACGTCTT |
